# Contribution of matrix metalloproteinases-1 genotypes to gastric cancer susceptibility in Taiwan

**DOI:** 10.1051/bmdcn/2017070203

**Published:** 2017-06-14

**Authors:** Mei-Due Yang, Kuo-Cheng Lin, Meng-Chun Lu, Long-Bin Jeng, Chieh-Lun Hsiao, Te-Cheng Yueh, Chun-Kai Fu, Hsin-Ting Li, Shiou-Ting Yen, Chia-Wen Lin, Cin-Wun Wu, Su-Yi Pang, Da-Tian Bau, Fuu-Jen Tsai

**Affiliations:** 1 Department of Clinical Nutrition, China Medical University Hospital Taichung 404 Taiwan; 2 Terry Fox Cancer Research Laboratory, China Medical University Hospital Taichung 404 Taiwan; 3 Graduate Institute of Biomedical Sciences, China Medical University Taichung 404 Taiwan; 4 Department of Medical Research, China Medical University Hospital Taichung 404 Taiwan

**Keywords:** Drinking, Gastric cancer, Genotype, MMP1, Polymorphism, Smoking, Taiwan

## Abstract

Expression of matrix metalloproteinase-1 (MMP1), an interstitial collagenase regulating the extracellular matrix, plays a major role in carcinogenesis of gastric cancer, a leading cause of death worldwide. In literature, the single-nucleotide polymorphism (SNP) promoter -1607 1G/2G (rs1799750) at the MMP1 gene promoter has been reported to alter its own transcription level. While the importance’s of the genotype of MMP1 promoter -1607 has not yet been studied in gastric cancer in Taiwan, our aim was to investigate MMP1 promoter -1607 genotypes and gastric cancer (GC) susceptibility in central Taiwan population. In the current hospital-based case-control study, the contribution of MMP1 promoter -1607 genotypes to GC risk was investigated among 121 GC patients and 363 gender- and age-matched healthy controls recruited and genotyped by the polymerase chain reaction-based restriction fragment length polymorphism (PCR-RFLP) methodology. We found that the genotypic and allelic frequencies were not differentially distributed between GC patient and control groups. The variant 1G containing genotypes have interactions with cigarrete smoking behaviors and *Helicobacter pylori* infection status, but not alcoholism on GC susceptibility determination. Our findings suggest that the variant 1G allele on MMP1 promoter -1607 may contribute to GC carcinogenesis and may be useful for GC early detection and prevention when combined with cigarrete smoking behaviors and *Helicobacter pylori* infection status.

## 1. Introduction

Gastric cancer (GC) is the fourth most common cancer and the second most frequent cause of death from cancer worldwide [1, 2]. Globally, it was estimated that about 800, 000 deaths occurred annually, and more than 70% of GC cases occurred in developed and developing countries and half of cases occurred in Eastern Asia, for instance, mainland China and Taiwan [2]. The incidence of GC varies among different countries as a result of genetic, epigenetic and environmental factors, which the accurate mechanisms for gastric carcinogenesis remained unknown. In addition to those known environmental factors such as unhealthy diets, infectious agents (*e.g*., *Helicobacter pylori)* and pre-existing conditions (*e.g*., pernicious anemia, atrophic gastritis, and intestinal polyps) [3, 4], the inherited genetic variations may play an important role in determining individual susceptibility to GC but are largely unrevealed, especially for the etiology of GC in Taiwan [5–12].

It is widely believed that GC carcinogenesis in a multi-cellular and multi-stage process in which the destruction of the extracellular microenvironment is a requisite for the transformation of normal tissues to tumors [13]. Hence, molecular investigations and understanding of the extracellular microenvironment and its deregulation during neoplasia is a key step to reveal the whole processes and mechanisms of malignancy. Matrix metalloproteinases (MMPs), also known as interstitial collagenases, are produced by both tumor and normal cells. MMPs may alter the microenvironment by degrading extracellular matrix, and subsequent cellular signals lead to the early stages of tumor formation [14]. Several of the MMPs have the unique activities to degrade the specific interstitial collagens *(e.g.* I, II, III, VII, VIII, X) and gelatin [15]. Among the various MMPs, MMP1 is the most ubiquitously expressed one [16] and its overexpression is associated with several specific pathological status, including inflammation, tumor invasion and metastasis [17]. The upregulation of MMP1 mRNA has been found in the tissues from the patients of various types of cancer, such as colorectal cancer, esophageal cancer and GC [18–22]. In addition, overexpression of MMP1 protein is associated with poor prognosis of esophageal cancer and colorectal cancer [20, 21]. This MMP1 overexpression may be attributed to the juxtaposition of transcription factor binding sites and cooperativity among the factors that bind to these sites within the promoter region of the MMP1 gene [23].

The most famous polymorphic site in the promoter region of MMP1 is rs1799750, which contains a guanine insertion/deletion polymorphism (1G/2G polymorphism) at position -1607 which generates the sequence 5’-GGA-3’ which has a 2G allele. The presence of a 2G polymorphism could have higher transcriptional activity of endogenous MMP1 than that with only one G because the guanine insertion creates a binding site for a member of the Ets transcription factor family. Clinically, the 2G allele was found to contribute to increased invasiveness of endometrial carcinomas, and to the development of ovarian cancer, lung cancer, and colorectal cancer [24–31].

Accordingly, we aimed at exploring whether the genotypes of MMP1 are associated with GC risk among Taiwanese. To test this hypothesis, we determined the genotypic frequency of three polymorphisms of the MMP1 gene at -1607 rs1799750 among a Taiwanese population, and analyzed its contribution to GC susceptibility and interactions with commonly known risk factors for GC, such as alcohol drinking, cigarette smoking, and *Helicobacter pylori* infection. To our knowledge, this is the first study carried out to evaluate the MMP1 genotypes in the high prevalence Taiwanese population.

## 2. Materials and methods

### 2.1. Study population and sample collection

One hundred and twenty one patients diagnosed with GC were recruited at the outpatient clinics of general surgery between 2005-2007 at the China Medical University Hospital, Taichung, Taiwan, Republic of China. The mean age of the gastric cancer patients were 51.26 (SD = 9.42) years. There were 56 females and 65 males. All patients voluntarily participated, completed a self-administered questionnaire and provided peripheral blood samples. Matched with age and gender, three hundreds and sixty-three non-cancer healthy people as controls were selected from the Health Examination Cohort of the hospital and the same questionnaires were recorded. Our study was approved by the Institutional Review Board of the China Medical University Hospital and written-informed consent was obtained from all participants with the help of Tissue Bank.

### 2.2. Genotyping assays

Genomic DNA was prepared from peripheral blood leucocytes using a QIAamp Blood Mini Kit (Blossom, Taipei, Taiwan). The forward and reverse primers for MMP1 promoter -1607 genotyping were 5’-TGACTTTTAAAACATAGTCTATGT-3’ and 5’- GATTGATTTGAGATAAGTCATAGC-3’, respectively. The polymerase chain reaction (PCR) cycling conditions were: one cycle at 94°C for 5 min; 35 cycles of 94°C for 30 s, 58°C for 30 s and 72°C for 30 s; and a final extension at 72°C for 10 min. After amplification, the PCR products were subject to digestion with *Alu I* restriction endonuclease for 2 h at 37°C and separation of 3% agarose gel electrophoresis. The genotypes were identified as homozygous 2G/2G (269 bp), heterozygous 1G/2G (269, 241 and 28 bp) and homozygous 1G/1G (241 and 28 bp). All the genotypic procession was repeated by two researchers independently and blindly as previously performed, with results being 100% concordant. All the processes in MMP1 promoter -1607 genotyping are much similar to the previous papers we published [32, 33].

### 2.3. Statistical analyses

Student’s t-test was used for the comparison of ages between the case and the control groups. Pearson’s Chi-square test was used to compare the distribution of the MMP1 promoter -1607 genotypes among the subgroups. The associations between the MMP1 promoter -1607 genotypes and GC risk were estimated by computing odds ratios (ORs) and their 95% confidence intervals (CIs) from logistic regression analysis. Any analyzing outcome with *P* < 0.05 was considered statistically significant.

## 3. Results

The selected characteristics of the GC patient group together with the control group are summarized in Table 1. The average BMI is of no difference (P > 0.05) between the control and GC patient groups. The percentage of alcohol consumers seemed to be higher in the GC patient group (32.2%) than that in the control group (23.1%), and the percentage of heavy drinkers are more than twice in the GC patient group (9.9%) than that in the control group (4.4%). As for the cigarette smoking habit analysis, there were significant trends that the GC patient group has higher percentage of cigarette consumers, especially heavy smokers, than the control group (34.7% *vs.* 19.6%, and 10.7% *vs.* 1.7%, respectively). As for the infection of *Helicobacter pylori,* 70.2% of the GC patients were positive, higher than 51.8% for the control subjects (P < 0.05). To sum up, the heavy consumption of alcohol and cigarette, in addition to the infection of *Helicobacter pylori*, are found to be the environmental factors contribute to increased GC risk in Taiwan.

**Table 1:** Selected Characteristics of the control and gastric cancer patient groups.

Character	Cases (n = 121)	Controls (n = 363)	*P*-value^a^
Age (SD)	51.3 (9.4)	53.2 (8.1)	0.8918
Gender (female/male)	56/65	168/195	1.0000
BMI average (SD)	27.1 (5.8)	26.7 (6.6)	0.9344
Alcohol consume			
Number (%)	39 (32.2)	84 (23.1)	0.0538
Heavy drinker (%)^b^	12(9.9)	11 (4.4)	0.0049*
Cigarette consume			
Number (%)	42 (34.7)	71 (19.6)	0.0012*
Heavy smoker (%)^c^	13 (10.7)	6(1.7)	0.0001*
H. pylori infection			
Number (%)	85 (70.2)	188 (51.8)	0.0005*
Tumor location			
Upper (%)	17 (14.0)		
Middle (%)	54 (44.6)		
Lower (%)	50 (41.3)		

a *P*-value based on *X*^2^ test.

b Drunken more than twice weekly or more than 100 *ml* per day for at least half year.

c More than 1 pack per day for at least half year.

The frequencies of the genotypes of MMP1 promoter -1607 polymorphisms in the GC patient and control groups are presented in Table 2. Compared with the 2G/2G genotype of MMP1 promoter -1607 as the reference group, there was no obvious increased risk in the 1G/2G or 1G/1G groups (OR = 0.88, 95% CI = 0.55-1.40, *P* = 0.5826; OR = 1.04, 95% CI = 0.60-1.79, *P* = 0.8970). The recessive and dominant models in the carrier comparison analysis showed a non-significant level for the variant 1G allele at MMP1 promoter -1607 to behave as a risk \determinant for GC (Table 2). The frequencies of the alleles for the MMP1 promoter -1607 polymorphism between GC patient and control groups are presented in Table 3. Supporting the findings in Table 2, the variant 1G allele at *XPD* codon 312 was not significantly associated with increased GC cancer risk (OR = 1.01, 95% CI = 0.75-1.35, *P* = 0.9702) (Table 3).

**Table 2:** Distribution of matrix metalloproteinase-1 (MMP1) promoter -1607 genotypes among the controls and patients with gastric cancer.

Genotype	Cases (n = 121)	Controls (n = 363)	Odds Ratio (95% CI)^a^	*P*-value^b^
MMP1 -1607				
2G/2G	43 (35.5)	123 (33.9)	1.00 (reference)	
1G/2G	49 (40.5)	160 (44.1)	0.88 (0.55-1.40)	0.5826
1G/1G	29 (24.0)	80 (22.0)	1.04 (0.60-1.79)	0.8970
*P*-value for trend				0.7821
				
Carrier comparison				
2G/2G + 1G/2G	92 (76.0)	283 (78.0)	1.00 (reference)	
1G/1G	29 (24.0)	80 (22.0)	1.12 (0.69-1.81)	0.6601
2G/2G	43 (35.5)	123 (33.9)	1.00 (reference)	
1G/1G + 1G/2G	78 (64.5)	240 (66.1)	0.93 (0.60-1.43)	0.7401

a CI, confidence interval;

b *P*-value based on *X*^2^ test without Yate’s correction.

**Table 3:** Allele frequencies for matrix metalloproteinase-1 (MMP1) promoter -1607 in the control and gastric cancer patient groups.

Allele	Cases (n = 242)	Controls (n = 726)	Odds Ratio (95% CI)^a^	*P*-value^a^
MMP1 -1607				
2G	135 (55.8)	406 (55.9)	1.00 (reference)	0.9702
1G	107 (44.2)	320 (44.1)	1.01 (0.75-1.35)	

a CI, confidence interval;

b *P*-value based on *X*^2^ test without Yate’s correction.

The genetic-environment interaction of genotype of MMP1 promoter -1607 and alcohol consumption for the risk of GC is presented in Table 4. Among those non-alcohol drinkers, the variant 1G allele could not increase the risk of gastric cancer (OR = 0.80, 95% CI = 0.48-1.32, *P* = 0.3866). The contribution of alcohol consumption behavior to gastric cancer risk was at a slightly increased level for those people without 1G allele at MMP1 promoter -1607 polymorphic site (OR = 1.22, 95% CI = 0.51-2.89, *P* = 0.6593), while for those with alcohol consumption behavior and 1G allele at promoter -1607, no synergistically increased moter -1607 gastric cancer risk was found (OR = 1.44, 95% CI = 0.80-2.58, *P* = 0.2223) (Table 4).

**Table 4:** Combined analysis of MMP1 promoter -1607 genotype and alcohol consumption for gastric cancer risk.

XPD codon 312 allele A carrier	Alcohol consumption	Controls/Cases	Odds Ratio (95% CI)^a^	*P*-value^b^
(-)	(-)	101/34	1.0 (reference)	
(+)	(-)	178/48	0.80 (0.48-1.32)	0.3866
(-)	(+)	22/9	1.22 (0.51-2.89)	0.6593
(+)	(+)	62/30	1.44 (0.80-2.58)	0.2223

a CI, confidence interval;

b *P*-value based on *X*^2^ test.

The genetic-environment interaction of genotype of MMP1 promoter -1607 and cigarette consumption for the risk of gastric cancer is presented in Table 5. Among those non-cigarette smokers, the variant 1G allele could slightly decrease the risk of gastric cancer (OR = 0.80, 95% CI = 0.48-1.33, *P* = 0.3911). The contribution of cigarette consumption behavior to gastric cancer risk was 1.77- and 1.96-fold for those people without (95% CI = 0.774.08, *P* = 0.1770) or with 1G allele at MMP1 promoter -1607 (95% CI = 1.08-3.55, *P* = 0.0265) (Table 5).

**Table 5:** Combined analysis of MMP1 promoter -1607 genotype and cigarette consumption for gastric cancer risk.

XPD codon 312 allele A carrier	Cigarette consumption	Controls/Cases	Odds Ratio (95% CI)^a^	*P*-value^b^
(-)	(-)	103/32	1.0 (reference)	
(+)	(-)	189/47	0.80 (0.48-1.33)	0.3911
(-)	(+)	20/11	1.77 (0.77-4.08)	0.1770
(+)	(+)	51/31	1.96 (1.08-3.55)*	0.0265*

a CI, confidence interval;

b *P*-value based on *X*^2^ test.

Among people not infected with *Helicobacter pylori*, the carriage of MMP1 promoter -1607 allele 1G was not associated with an decreased risk of gastric cancer (OR = 0.84, 95% CI = 0.41-1.73, *P* = 0.6387). On the contrary, *Helicobacter pylori* infection was associated with an increased risk of gastric cancer among those without variant 1G allele of MMP1 promoter -1607 (OR = 2.58, 95% CI = 1.27-5.26, *P* = 0.0078). At the same time, *Helicobacter* pylori-infected individuals who were carriers of MMP1 promoter -1607 allele 1G also exhibited an increased risk of gastric cancer (OR = 1.84, 95% CI = 1.02-3.33, *P* = 0.0423). In summary, the results in Table 4, 5 and 6 indicated a synergistic interaction of MMP1 promoter -1607 allele 1G with cigarette smoking and *Helicobacter pylori* infection, but not with alcohol drinking, in the development of gastric cancer.

**Table 6:** Combined analysis of MMP1 promoter -1607 genotype and *H. pylori* infection for gastric cancer risk.

XPD codon 312 allele A carrier	H. pylori infection	Controls/ Cases	Odds Ratio (95% CI)l^a^	P-value^b^
(-)	(-)	80/18	1.0 (reference)	
(+)	(-)	95/18	0.84 (0.41-1.73)	0.6387
(-)	(+)	43/25	2.58 (1.27-5.26)*	0.0078*
(+)	(+)	145/60	1.84 (1.02-3.33)*	0.0423*

a CI, confidence interval;

b *P*-vaue based on *X*^2^ test

## 4. Discussion

In the present study, we have investigated the association of MMP1 promoter -1607 genotypes, with gastric cancer susceptibility in Taiwan. The results demonstrated that the MMP1 promoter -1607 genotypes were not significantly associated with risk of developing gastric cancer in Taiwan (Tables 2, 3). To the best of our knowledge, this is the first epidemiology study based on molecular genetics to find the significant association between MMP1 genotypes and the susceptibility to gastric cancer with the analysis of the gene-environment interaction in Taiwan. Interestingly, a synergistic interaction of MMP1 promoter -1607 allele 1G with cigarette smoking (Table 5) and *Helicobacter pylori* infection (Table 6), but not with alcohol drinking (Table 4), in the development of gastric cancer. In 2004, Matsumura and his coworkers firstly examined the contribution of MMP1 genotypes to GC risk [34], but findings that the genotypes were neither associated with the GC risk nor the prognosis such as lymph node metastasis and clinical stages. From that time, a few reports focused on investigating the associations of three common MMP1 polymorphism, promoter -1607, with GC risk among different ethnicities, but with conflicting and inclusive results [35–38]. We have summarized the characteristics of each of the literature in the last Table of this article, in addition to our current findings (Table 7).

**Table 7:** The characteristics of individual reports investigating the genotypes of MMP1 promoter -1607 to gastric cancer risk.

Author	Year	Population	Control, n	Case, n	Major findings
Mtsumura	2004	Japanese	166	215	MMP1 -1607 was not associated with GC risk, but may be markers for prognosis prediction such as lymph node metastasis and clinical stages.
Jin	2005	Mainland China	350	183	MMP1 -1607 was neither associated with GC risk nor prognostic indexes such as metastasis.
Fang	2010	Mainland China	252	246	There were no significant difference in the genotype or allele frequency of the MMP-1 16071G/2G between the case and control groups.
Devulapalli	2014	India	202	166	MMP1 -1607 was a determinant for GC risk, especially for those male gender, > 50 years, addicted to alcohol and smoking.
Dey	2014	India	145	145	MMP1 -1607 was not associated with GC risk. Other promoter SNPs on MMP1 may be markers for prognosis prediction such as metastasis.
Yang	current	Taiwanese	363	121	MMP1 -1607 was not associated with GC risk. It may interact with smoking and HP infection status determine the GC susceptibility.

In literature, there were a few studies providing evidence for the increased risk of GC among cigarette smokers [9, 39–43], but some others were not [44, 45]. In the current study from the epidemiologic viewpoint, we have also found that cigarette smoking may also contribute to the risk of GC (P = 0.0012), especially for those heavy smokers (P = 0.0001) (Table 1). Similarly, we have found that cigarette consumption behavior among those carrying the 1G allele at MMP1 promoter -1607 were of 1.96-fold (95% CI = 1.08-3.55, *P* = 0.0265) increased risk of developing GC. In 2012, Smyth and his colleagues have investigated the contribution of tobacco usage history to their 5-year survival status, finding that smoking was a risk factor of gastric cancer and associated with worse 5-year survival [46]. For those who smoked less than 20 pack-years (defined as light smokers) and equal to or more than 20 pack-years (defined as heavy smokers), their GC disease-specific survival, 5-year disease-free survival and overall survival rates were less than the non-smokers [46]. To sum up, the behavior of cigarette smoking may not only contribute to individual GC risk, but to overall death rates after the undergoing of surgical resection. The detail interaction of MMP1 genotype with smoking behavior on GC etiology needs further investigations.

As for *Helicobacter pylori* infection, the data in Table 1 showed that about half (51.8%) of the Taiwanese people were infected, which were significantly lower than 70.2% in the gastric cancer patients (P = 0.0005) (Table 1). The stratified analysis showed that among those people without the 1G allele at MMP1 promoter -1607, the status of *Helicobacter pylori* infection has caused a significant higher risk of GC to them (OR = 2.58, 95% CI = 1.27-5.26, *P* = 0.0078) (Table 6). The *Helicobacter pylori* infection would perform an increase of GC risk for those people with the 1G allele at MMP1 promoter -1607 (OR = 1.84, 95% CI = 1.02-3.33, *P* = 0.0423) from those people without *Helicobacter pylori* infection (OR = 0.84, 95% CI = 0.41-1.73, *P* = 0.6387). Thus, the MMP1 may cause an alteration of extracellular microenvironment, interact with the consequence of *Helicobacter pylori* infection, and determine the GC initiation and development. The detail mechanism needs further investigations.

These results suggested that genetic variants of MMP1 promoter -1607 may play a critical role in GC etiology indirectly *via* the alteration of extracellular matrix components, and *Helicobacter pylori* infection status. In conclusion, our findings suggest that although the MMP1 promoter -1607 genotype itself was not associated with risk to GC, but the 1G allele of MMP1 promoter -1607 is still an useful marker combined with cigarette smoking, and *Helicobacter pylori* infection status, for individualized early detection, prevention and anticancer intervention.

## Conflict of interest

The authors declare that they have no conflict of interest.

## References

[1] Parkin DM. International variation. Oncogene 2004; 23: 6329–6340. 15322508 10.1038/sj.onc.1207726

[2] Ferlay J, Shin HR, Bray F, Forman D, Mathers C, Parkin DM. Estimates of worldwide burden of cancer in 2008: GLOBOCAN 2008. Int J Cancer. 2010; 127: 2893–2917. 21351269 10.1002/ijc.25516

[3] Lee SG, Kim B, Choi J, Kim C, Lee I, Song K. Genetic polymorphisms of XRCC1 and risk of gastric cancer. Cancer Lett. 2002; 187: 53–60. 12359351 10.1016/s0304-3835(02)00381-6

[4] Gastric cancer and Helicobacter pylori: a combined analysis of 12 case control studies nested within prospective cohorts. Gut. 2001; 49: 347–53. 11511555 10.1136/gut.49.3.347 PMC1728434

[5] Fuchs CS, Mayer RJ. Gastric carcinoma. N Engl J Med. 1995; 333: 32–41. 7776992 10.1056/NEJM199507063330107

[6] Lin CH, Lin CC, Tsai CW, Chang WS, Yang CW, Bau DT. Association of caveolin-1 genotypes with gastric cancer in Taiwan. Anticancer Res. 2014; 34: 2263–2267. 24778029

[7] Kuo WH, Huang CY, Fu CK, Hsieh YH, Liao CH, Hsu CM, et al Effects of interleukin-10 polymorphisms and smoking on the risk of gastric cancer in Taiwan. In Vivo. 2014; 28: 967–971. 25189915

[8] Kuo HW, Huang CY, Fu CK, Liao CH, Hsieh YH, Hsu CM, et al The significant association of CCND1 genotypes with gastric cancer in Taiwan. Anticancer Res. 2014; 34: 4963–4968. 25202078

[9] Ji HX, Chang WS, Tsai CW, Wang JY, Huang NK, Lee AS, et al Contribution of DNA Repair Xeroderma Pigmentosum Group D Genotype to Gastric Cancer Risk in Taiwan. Anticancer Res. 2015; 35: 4975–4981. 26254397

[10] Yang MD, Wang HC, Chang WS, Tsai CW, Bau DT. Genetic polymorphisms of DNA double strand break gene Ku70 and gastric cancer in Taiwan. BMC Cancer. 2011; 11: 174. 21575261 10.1186/1471-2407-11-174 PMC3111404

[11] Bau DT, Wang HC, Liu CS, Chang CL, Chiang SY, Wang RF, et al Single-nucleotide polymorphism of the Exo1 gene: association with gastric cancer susceptibility and interaction with smoking in Taiwan. Chin J Physiol. 2009; 52: 411–418. 20337148 10.4077/cjp.2009.amh076

[12] Chiu CF, Wang CH, Wang CL, Lin CC, Hsu NY, Weng JR, et al A novel single nucleotide polymorphism in XRCC4 gene is associated with gastric cancer susceptibility in Taiwan. Ann Surg Oncol. 2008; 15: 514–518. 17987338 10.1245/s10434-007-9674-3

[13] Park CC, Bissell MJ, Barcellos-Hoff MH. The influence of the microenvironment on the malignant phenotype. Mol Med Today. 2000; 6: 324–329. 10904250 10.1016/s1357-4310(00)01756-1

[14] Lukashev ME, Werb Z. ECM signalling: orchestrating cell behaviour and misbehaviour. Trends Cell Biol. 1998; 8: 437–441. 9854310 10.1016/s0962-8924(98)01362-2

[15] Birkedal-Hansen H, Moore WG, Bodden MK, Windsor LJ, Birkedal-Hansen B, DeCarlo A, et al Matrix metalloproteinases: a review. Crit Rev Oral Biol Med. 1993; 4: 197–250. 8435466 10.1177/10454411930040020401

[16] Vincenti MP, White LA, Schroen DJ, Benbow U, Brinckerhoff CE. Regulating expression of the gene for matrix metalloproteinase-1 (collagenase): mechanisms that control enzyme activity, transcription, and mRNA stability. Crit Rev Eukaryot Gene Expr. 1996; 6: 391–411. 8959374 10.1615/critreveukargeneexpr.v6.i4.40

[17] Pulukuri SM, Rao JS. Matrix metalloproteinase-1 promotes prostate tumor growth and metastasis. Int J Oncol. 2008; 32: 757–765. 18360703 PMC2292413

[18] Chambers AF, Matrisian LM. Changing views of the role of matrix metalloproteinases in metastasis. J Natl Cancer Inst. 1997; 89: 1260–1270. 9293916 10.1093/jnci/89.17.1260

[19] Hewitt RE, Leach IH, Powe DG, Clark IM, Cawston TE, Turner DR. Distribution of collagenase and tissue inhibitor of metalloproteinases (TIMP) in colorectal tumours. Int J Cancer. 1991; 49: 666–672. 1657796 10.1002/ijc.2910490507

[20] Murray GI, Duncan ME, Arbuckle E, Melvin WT, Fothergill JE. Matrix metalloproteinases and their inhibitors in gastric cancer. Gut. 1998; 43: 791–797. 9824606 10.1136/gut.43.6.791 PMC1727341

[21] Murray GI, Duncan ME, O’Neil P, McKay JA, Melvin WT, Fothergill JE. Matrix metalloproteinase-1 is associated with poor prognosis in oesophageal cancer. J Pathol. 1998; 185: 256–261. 9771478 10.1002/(SICI)1096-9896(199807)185:3<256::AID-PATH115>3.0.CO;2-A

[22] Baker EA, Leaper DJ. The plasminogen activator and matrix metalloproteinase systems in colorectal cancer: relationship to tumour pathology. Eur J Cancer. 2003; 39: 981–988. 12706368 10.1016/s0959-8049(03)00065-0

[23] Rutter JL, Mitchell TI, Buttice G, Meyers J, Gusella JF, Ozelius LJ, et al A single nucleotide polymorphism in the matrix metalloproteinase-1 promoter creates an Ets binding site and augments transcription. Cancer Res. 1998; 58: 5321–5325. 9850057

[24] Nishioka Y, Kobayashi K, Sagae S, Ishioka S, Nishikawa A, Matsushima M, et al A single nucleotide polymorphism in the matrix metalloproteinase-1 promoter in endometrial carcinomas. Jpn J Cancer Res. 2000; 91: 612–615. 10874213 10.1111/j.1349-7006.2000.tb00989.x PMC5926401

[25] Kanamori Y, Matsushima M, Minaguchi T, Kobayashi K, Sagae S, Kudo R, et al Correlation between expression of the matrix metalloproteinase-1 gene in ovarian cancers and an insertion/deletion polymorphism in its promoter region. Cancer Res. 1999; 59: 4225–4227. 10485461

[26] Ghilardi G, Biondi ML, Mangoni J, Leviti S, DeMonti M, Guagnellini E, et al Matrix metalloproteinase-1 promoter polymorphism 1G/2G is correlated with colorectal cancer invasiveness. Clin Cancer Res. 2001; 7: 2344–2346. 11489811

[27] Hinoda Y, Okayama N, Takano N, Fujimura K, Suehiro Y, Hamanaka Y, et al Association of functional polymorphisms of matrix metalloproteinase (MMP)-1 and MMP-3 genes with colorectal cancer. Int J Cancer. 2002; 102: 526–529. 12432557 10.1002/ijc.10750

[28] Zinzindohoue F, Lecomte T, Ferraz JM, Houllier AM, Cugnenc PH, Berger A, et al Prognostic significance of MMP-1 and MMP-3 functional promoter polymorphisms in colorectal cancer. Clin Cancer Res. 2005; 11: 594–599. 15701845

[29] Elander N, Soderkvist P, Fransen K. Matrix metalloproteinase (MMP) -1, -2, -3 and -9 promoter polymorphisms in colorectal cancer. Anticancer Res. 2006; 26: 791–795. 16739355

[30] Hettiaratchi A, Hawkins NJ, McKenzie G, Ward RL, Hunt JE, Wakefield D, et al The collagenase-1 (MMP-1) gene promoter polymorphism - 1607/2G is associated with favourable prognosis in patients with colorectal cancer. Br J Cancer. 2007; 96: 783–792. 17311017 10.1038/sj.bjc.6603630 PMC2360084

[31] Zhu Y, Spitz MR, Lei L, Mills GB, Wu X. A single nucleotide polymorphism in the matrix metalloproteinase-1 promoter enhances lung cancer susceptibility. Cancer Res. 2001; 61: 7825–7829. 11691799

[32] Tsai CW, Chang WS, Gong CL, Shih LC, Chen LY, Lin EY, et al Contribution of Matrix Metallopeptidase-1 Genotypes, Smoking, Alcohol Drinking and Areca Chewing to Nasopharyngeal Carcinoma Susceptibility. Anticancer Res. 2016; 36: 3335–3340. 27354591

[33] Sun KT, Tsai CW, Chang WS, Shih LC, Chen LY, Tsai MH, et al The Contribution of Matrix Metalloproteinase-1 Genotype to Oral Cancer Susceptibility in Taiwan. In Vivo. 2016; 30: 439–444. 27381606

[34] Matsumura S, Oue N, Kitadai Y, Chayama K, Yoshida K, Yamaguchi Y, et al A single nucleotide polymorphism in the MMP-1 promoter is correlated with histological differentiation of gastric cancer. J Cancer Res Clin Oncol. 2004; 130: 259–265. 14986114 10.1007/s00432-004-0543-1 PMC12161842

[35] Jin X, Kuang G, Wei LZ, Li Y, Wang R, Guo W, et al No association of the matrix metalloproteinase 1 promoter polymorphism with susceptibility to esophageal squamous cell carcinoma and gastric cardiac adenocarcinoma in northern China. World J Gastroenterol. 2005; 11: 2385–2389. 15832405 10.3748/wjg.v11.i16.2385 PMC4305622

[36] Fang WL, Liang WB, Gao LB, Zhou B, Xiao FL, Zhang L. Genetic polymorphisms in Matrix Metalloproteinases -1 and -7 and susceptibility to gastric cancer: an association study and meta-analysis. Iran J Allergy Asthma Immunol. 2013; 12: 203–210. 23893803

[37] Devulapalli K, Bhayal AC, Porike SK, Macherla R, Akka J, Nallari P., et al Role of interstitial collagenase gene promoter polymorphism in the etiology of gastric cancer. Saudi J Gastroenterol. 2014; 20: 309–314. 25253367 10.4103/1319-3767.141693 PMC4196347

[38] Dey S, Ghosh N, Saha D, Kesh K, Gupta A, Swarnakar S. Matrix metalloproteinase-1 (MMP-1) Promoter polymorphisms are well1 linked with lower stomach tumor formation in eastern Indian population. PLoS One. 2014; 9: e88040. 24505369 10.1371/journal.pone.0088040 PMC3914871

[39] Chow WH, Swanson CA, Lissowska J, Groves FD, Sobin LH, Nasierowska-Guttmejer A, et al Risk of stomach cancer in relation to consumption of cigarettes, alcohol, tea and coffee in Warsaw, Poland. Int J Cancer. 1999; 81: 871–876. 10362132 10.1002/(sici)1097-0215(19990611)81:6<871::aid-ijc6>3.0.co;2-#

[40] Gammon MD, Schoenberg JB, Ahsan H, Risch HA, Vaughan TL, Chow WH, et al Tobacco, alcohol, and socioeconomic status and denocarcinomas of the esophagus and gastric cardia. J Natl Cancer Inst. 1997; 89: 1277–1284. 9293918 10.1093/jnci/89.17.1277

[41] Inoue M, Tajima K, Hirose K, Kuroishi T, Gao CM, Kitoh T. Lifestyle and subsite of gastric cancer–joint effect of smoking and drinking habits. Int J Cancer. 1994; 56: 494–499. 8112885 10.1002/ijc.2910560407

[42] Sasazuki S, Sasaki S, Tsugane S. Cigarette smoking, alcohol consumption and subsequent gastric cancer risk by subsite and histologic type. Int J Cancer. 2002; 101: 560–566. 12237898 10.1002/ijc.10649

[43] Tredaniel J, Boffetta P, Buiatti E, Saracci R, Hirsch A. Tobacco smoking and gastric cancer: review and meta-analysis. Int J Cancer. 1997; 72: 565–573. 9259392 10.1002/(sici)1097-0215(19970807)72:4<565::aid-ijc3>3.0.co;2-o

[44] Moy KA, Fan Y, Wang R, Gao YT, Yu MC, Yuan JM. Alcohol and tobacco use in relation to gastric cancer: a prospective study of men in Shanghai, China. Cancer Epidemiol Biomarkers Prev. 2010; 19: 2287–2297. 20699372 10.1158/1055-9965.EPI-10-0362 PMC2936659

[45] Engeland A, Andersen A, Haldorsen T, Tretli S. Smoking habits and risk of cancers other than lung cancer: 28 years’ follow-up of 26, 000 Norwegian men and women. Cancer Causes Control. 1996; 7: 497506. 10.1007/BF00051881 8877046

[46] Smyth EC, Capanu M, Janjigian YY, Kelsen DK, Coit D, Strong VE, et al Tobacco use is associated with increased recurrence and death from gastric cancer. Ann Surg Oncol. 2012; 19: 2088–2094. 22395977 10.1245/s10434-012-2230-9

